# Association between homocysteine and non-alcoholic fatty liver disease in Chinese adults: a cross-sectional study

**DOI:** 10.1186/s12937-016-0221-6

**Published:** 2016-12-12

**Authors:** Haijiang Dai, Weijun Wang, Xiaohong Tang, Ruifang Chen, Zhiheng Chen, Yao Lu, Hong Yuan

**Affiliations:** 1 Center of Clinical Pharmacology, the Third Xiangya Hospital, Central South University, 138 Tong-Zi-Po Road, Changsha, Hunan Zip 410013 People’s Republic of China; 2 Center of Health Management, the Third Xiangya Hospital, Central South University, Changsha, Hunan Province Zip 410013 People’s Republic of China; 3 Division of Gastroenterology, Union Hospital, Huazhong University of Science and Technology, Wuhan, Hubei Province Zip 430022 People’s Republic of China

**Keywords:** Homocysteine, Non-alcoholic fatty liver disease, Gender, Body mass index, Interaction

## Abstract

**Background:**

Non-alcoholic fatty liver disease (NAFLD) is the most common liver disease worldwide, and its prevalence is likely to rise even further. To help understand the pathogenesis and early prevention of progressive NAFLD, this large-scale study was designed to explore the potential association between homocysteine and the prevalence of NAFLD.

**Methods:**

A total of 7203 subjects aged 18 years or older were enrolled in this cross-sectional study. The association of homocysteine with the prevalence of NAFLD, in the total sample and stratified by subgroups, was examined using multiple logistic regression analyses.

**Results:**

Subjects in the higher quartiles of homocysteine had a higher prevalence of NAFLD. After multivariate adjustment, the odds ratio (OR) for NAFLD in the highest compared with the lowest quartile of homocysteine was 2.08 (95% confidence interval [CI] 1.61, 2.67). Moreover, in the subgroup analyses, we found an effect modification by gender, body mass index (BMI) and smoking status on the association between homocysteine and the prevalence of NAFLD (*P* for interaction: 0.001, 0.002 and <0.001, respectively). A stronger association was observed in female, obese and non-smoking adults than in male, normal weight and smoking subjects.

**Conclusion:**

Homocysteine was significantly associated with the prevalence of NAFLD, particularly in female, obese or non-smoking adults.

## Background

Non-alcoholic fatty liver disease (NAFLD), which encompasses a spectrum of conditions associated with lipid deposition in hepatocytes, is the most common liver disease. Worldwide, the overall prevalence of NAFLD diagnosed by imaging was 25.2% with the highest prevalence rates were reported from South America (30.5%) and the Middle East (31.8%) [1]. In China, NAFLD affects over a quarter of the population, and its prevalence is still increasing rapidly as a result of considerable changes in lifestyle and aging [2–4]. NAFLD is often associated with metabolic risk factors, such as obesity, type two diabetes, dyslipidemia, and insulin resistance. In addition to hepatic complications, NAFLD is also associated with serious systemic consequences. NAFLD has been widely accepted to significantly increase the morbidity and mortality of cardiovascular diseases [5–7].

Homocysteine is a sulfhydryl-containing amino acid mainly produced and catabolized in the liver [8, 9]. A growing body of evidence shows that homocysteine mediates cardiovascular problems by its adverse effects on cardiovascular endothelium and smooth muscle cells [10]. Moreover, homocysteine can alter intracellular lipid metabolism and may promote hepatic fat accumulation [11, 12]. Thus, it is plausible that homocysteine could be an effective target for preventing the progression of NAFLD and its related cardiovascular complications. However, only a few studies have explored the association between homocysteine and the prevalence of NAFLD, and the answer remains controversial [13–15]. In addition, it is also important to confirm whether these associations are moderated by other closely related factors, such as gender and body mass index (BMI), as substantial differences exist with regard to the metabolism of homocysteine between males and females as well as among BMI groups [10, 16].

In this study, we performed a cross-sectional analysis to determine whether elevated homocysteine was associated with an increased prevalence of NAFLD in Chinese adults. Moreover, we also explored the possible effect modification by other related factors on the association between homocysteine and the prevalence of NAFLD.

## Methods

### Study population

The Health Management Center of Third Xiangya Hospital is one of China’s largest examination centers, mainly servicing individuals from hundreds of institutions in Changsha. In this cross-sectional study, we consecutively recruited 13,916 individuals who underwent a health examination in the center from January 2014 to December 2014. The inclusion criteria were: 1) aged 18 years or older; 2) availability of abdominal ultrasonography examination; 3) and undergoing plasma homocysteine measurements. Questionnaires were answered by all of the enrolled subjects to collect information on medical history, alcohol consumption, cigarette smoking, etc. In order to remove the effect of alcohol on the fatty liver, we excluded subjects with excessive alcohol consumption (*n* = 1629) as well as those with incomplete information on alcohol consumption (*n* = 4451). As a result, 7836 subjects were included. Of these kept subjects, 565 subjects were diagnosed or self-reported to have viral hepatitis, schistosomiasis liver disease or other chronic liver diseases, 9 subjects were self-reported taking steatogenic medications, 12 subjects were self-reported taking vitamin B or folic acid, and 47 subjects had no available data on height or body weight; all of these patients were excluded from our study. Finally, 7203 subjects were screened and deemed eligible for the present study.

This study was approved by the Medical Ethics Committee of Third Xiangya Hospital. All of the experiments in this study were performed according to the guidelines from the Helsinki Declaration, and written informed consent was obtained from all participants.

### Data collection and measurements

Information on age, gender, alcohol consumption, cigarette smoking, physical activity, education level, and medical history were obtained from standardized questionnaires through face-to-face interviews. Alcohol consumption was evaluated with questions regarding the types of alcoholic beverages, the frequency of alcohol consumption per week and the usual amount consumed per occasion. Subjects who reported alcohol consumption ≥140 g/week for men and ≥70 g/week for women were deemed to have excessive alcohol consumption [17]. Smoking was recorded as daily (at least one cigarette/day), occasional (less than one cigarette/day), former (having quit for at least 6 months), or never smoking. For our analyses, only two categories were considered: current smoking (daily and occasional smoking) and non-smoking (never and former smoking) [18]. Physical activity was defined as the frequency of physical activity during leisure time and was scored as inactive (1–2 times per week), moderate (3–5 times per week), or active (≥6 times per week).

Standing height and body weight were measured without shoes or thick clothing, and BMI was calculated as body weight in kilograms divided by height in meters squared. Blood pressure (BP) was measured in the sitting position after a 10-min rest period using an appropriately sized cuff and a corrected mercury sphygmomanometer. Systolic BP and diastolic BP were each measured twice in this study, with a 30 s interval. If the two measurements differed by >5 mmHg, BP was re-measured. Finally, the BP was calculated as the average of the three measurements. Hypertension was defined by the presence of any of the following: systolic BP ≥140 mmHg and/or diastolic BP ≥90 mmHg, a history of hypertension, or current use of antihypertensive agents.

Venous blood sampling was performed after overnight fasting for 8–12 h, and the blood glucose, alanine transaminase (ALT), total bilirubin (TBIL), albumin (ALB), platelet count (PLT), serum uric acid, triglyceride (TG), total cholesterol (TC), high-density lipoprotein cholesterol (HDL-C), high-sensitivity C-reactive protein (hs-CRP), serum creatinine and homocysteine levels were determined using standard laboratory methods. The laboratory methods were consistent throughout the study period. All blood samples were tested using an auto-analyzer (Hitachi 7600-110; Hitachi, Tokyo, Japan) at the central laboratory of Third Xiangya Hospital. Diabetes was diagnosed by fasting blood glucose ≥7.0 mmol/L, a history of diabetes, or current use of hypoglycemic agents [19].

### Definition of non-alcoholic fatty liver disease

Hepatic steatosis was diagnosed upon abdominal ultrasonography by experienced and trained radiologists who were blinded to the subjects’ clinical diagnoses and biochemical tests. Positive abdominal ultrasound images included: diffusely increased liver near field ultrasound echo (‘bright liver’), liver echo greater than kidney, vascular blurring and the gradual attenuation of far field ultrasound echo. Subjects with at least two of the abnormal findings listed above were diagnosed with hepatic steatosis [17, 20]. Because all of the subjects with secondary causes for hepatic steatosis such as excessive alcohol consumption, viral hepatitis, or use of steatogenic medication were excluded from our study, NAFLD was defined by the presence of hepatic steatosis [20, 21].

### Statistical analysis

In order to derive a deeper understanding of the relationship between serum homocysteine levels and the prevalence of NAFLD, all of the study subjects were classified into four groups by their quartiles of homocysteine (Q1: <5.1 μmol/L, Q2: 5.1 to <7.1 μmol/L, Q3: 7.1 to <9.9 μmol/L, Q4: ≥9.9 μmol/L). Basic characteristics of the study subjects were presented as the mean ± standard deviation (SD) or median (interquartile range) for continuous variables and as numbers with percentages for categorical variables. Variables that displayed a skewed distribution (age, ALT, TBIL, TG, hs-CRP and homocysteine) were log transformed to normal before analysis.

For continuous variables, one-way ANOVA with a post-hoc *t*-test with Bonferroni correction for multiple comparisons was used to compare differences between the quartiles of serum homocysteine. For categorical variables, differences between group frequencies were assessed with the Pearson χ^2^ test. The odds ratio (OR) with 95% confidence intervals (CIs) were calculated using logistic regression to determine the risk of NAFLD for each quartile of homocysteine, with the lowest quartile regarded as the reference category. Moreover, age, gender, BMI (categorized as normal: <24 kg/m^2^, overweight: 24 to <28 kg/m^2^, and obesity: ≥28 kg/m^2^, as based on the Working Group on Obesity in China guidelines [22]), current smoking, hypertension, and diabetes were evaluated to assess whether there was any significant interaction between these variables and the relationship between homocysteine levels and the prevalence of NAFLD.

All of the analyses were performed using IBM SPSS Statistics Ver. 22.0 (IBM Co., Armonk, NY, USA), and *P* < 0.05 was considered to indicate statistical significance. Post-hoc Bonferroni correction was used for multiple comparisons.

## Results

### Characteristics of the study subjects

Of the 7203 subjects, 2370 (32.9%) were diagnosed with NAFLD, and the mean homocysteine level was 7.0 (5.0, 9.9) μmol/L. The characteristics of the study population in total and according to homocysteine quartiles are displayed in Table 1. When compared with subjects in Q1 group, those with higher homocysteine levels were more likely to be older, male, and current smokers, and they tended to have increased prevalence of hypertension as well as higher levels of BMI, ALT, TBIL, ALB, serum uric acid, TG, hs-CRP and creatinine. In addition, the frequency of physical activity, prevalence of drinking and diabetes, and levels of education, PLT, TC, and HDL-C were also significantly different among the homocysteine quartiles.

**Table 1:** Characteristics of the study population in total and according to homocysteine quartiles

Variables	Total	Quartiles of Homocysteine				*P* value
		Q1 (<5.1)	Q2 (5.1 to <7.1)	Q3 (7.1 to <9.9)	Q4 (≥9.9)	
*N*	7203	1876	1815	1697	1815	
Homocysteine (μmol/L)^a^	7.0 (5.0, 9.9)	4.0 (3.9, 5.0)	6.0 (5.9, 7.0)^#^	8.3 (7.9, 9.0)^#^	12.3 (10.8, 14.9)^#^	<0.001
Age (years)^a^	49.0 (40.0, 59.0)	46.0 (38.0, 52.0)	49.0 (40.0, 57.0)^#^	48.0 (39.0, 58.0)^#^	53.0 (42.0, 70.0)^#^	<0.001
Male, %	3801 (52.8)	477 (25.4)	794 (43.7)^#^	1024 (60.3)^#^	1506 (83.0)^#^	<0.001
BMI (kg/m^2^)	24.1 ± 3.3	23.6 ± 3.2	24.0 ± 3.2^#^	24.2 ± 3.2^#^	24.7 ± 3.3^#^	<0.001
Current smoker, %	1698 (24.0)	284 (15.5)	364 (20.6)^#^	439 (26.2)^#^	611 (33.9)^#^	<0.001
Physical activity						<0.001
Inactive, %	3590 (53.6)	991 (58.7)	888 (54.0)	898 (55.6)	813 (46.4)	
Moderate, %	1769 (26.4)	461 (27.3)	472 (28.7)	408 (25.2)	428 (24.4)	
Active, %	1342 (20.0)	235 (13.9)	284 (17.3)	310 (19.2)	513 (29.2)	
Education						<0.001
Illiteracy/Primary, %	282 (4.1)	74 (4.2)	62 (3.6)	72 (4.4)	74 (4.2)	
Middle school, %	2234 (32.5)	683 (38.3)	612 (35.8)	479 (29.4)	460 (26.2)	
College or higher, %	4361 (63.4)	1026 (57.5)	1035 (60.6)	1081 (66.2)	1219 (69.5)	
Drinking, %	1459 (20.3)	252 (13.4)	276 (15.2)	412 (24.3)^#^	519 (28.6)^#^	<0.001
Hypertension, %	2144 (30. 0)	342 (18.4)	485 (27.0)^#^	507 (30.1)^#^	810 (45.0)^#^	<0.001
Diabetes, %	543 (7.5)	115 (6.1)	132 (7.3)	127 (7.5)	169 (9.3)^#^	0.002
ALT (U/L)^a^	21.0 (15.0, 30.0)	18.0 (14.0, 26.0)	20.0 (15.0, 29.0)^#^	22.0 (17.0, 33.0)^#^	23.0 (17.0, 33.0)^#^	<0.001
TBIL (μmol/L)^a^	14.8 (12.1, 18.3)	14.1 (11.6, 17.1)	14.7 (11.9, 18.2)^#^	15.1 (12.3, 18.8)^#^	15.6 (12.5, 19.1)^#^	<0.001
ALB (g/L)	46.5 ± 2.6	46.2 ± 2.5	46.6 ± 2.5^#^	46.8 ± 2.6^#^	46.5 ± 2.8^#^	<0.001
PLT (10^9^/L)	213.1 ± 55.0	218.2 ± 55.0	215.8 ± 55.5	215.5 ± 56.7	202.9 ± 51.6^#^	<0.001
Uric acid (μmol/L)	297.7 ± 92.2	263.8 ± 84.5	289.6 ± 91.9^#^	300.5 ± 86.0^#^	338.3 ± 90.0^#^	<0.001
TG (mmol/L)^a^	1.3 (0.9, 1.9)	1.2 (0.8, 1.7)	1.3 (0.9, 1.8)^#^	1.3 (0.9, 2.0)^#^	1.4 (1.0, 2.0)^#^	<0.001
TC (mmol/L)	5.2 ± 1.0	5.1 ± 1.0	5.2 ± 1.0^#^	5.2 ± 1.0^#^	5.1 ± 1.0	0.001
HDL-C (mmol/L)	1.6 ± 0.4	1.7 ± 0.4	1.6 ± 0.4^#^	1.6 ± 0.4^#^	1.5 ± 0.4^#^	<0.001
hs-CRP (mg/L)^a^	1.1 (0.6, 1.9)	0.9 (0.5, 1.7)	1.1 (0.6, 2.0)^#^	1.1 (0.6, 2.0)^#^	1.2 (0.7, 2.1)^#^	<0.001
Creatinine (μmol/L)	67.2 ± 22.0	57.4 ± 12.2	64.6 ± 18.9^#^	67.9 ± 15.0^#^	79.4 ± 30.7^#^	<0.001
Prevalence of NAFLD, %	2370 (32.9)	372 (19.8)	513 (28.3)^#^	640 (37.7)^#^	845 (46.6)^#^	<0.001

*Abbreviations*: *BMI* body mass index, *ALT* alanine transaminase, *TBIL* total bilirubin, *ALB* albumin, *PLT* platelet count, *TG* triglyceride, *TC* total cholesterol, *HDL-C* high-density lipoprotein cholesterol, *hs-CRP* high-sensitivity C-reactive protein

^a^Variables were log transformed before analysis

^#^ *P* <0.05, in comparison with the reference group (quartile 1). *P* values were corrected by Bonferroni’s method due to multiple testing

### Association between homocysteine and non-alcoholic fatty liver disease

As shown in Table 1, the prevalence of NAFLD progressively increased in the higher quartiles of homocysteine (19.8, 28.3, 37.7, and 46.6%, respectively). Table 2 shows the multiple adjusted association between the quartiles of homocysteine and NAFLD. In the adjusted model 1, after adjustment for age, the OR for NAFLD in the highest compared to the lowest quartile of homocysteine was 3.10 (95% CI 2.67, 3.59). After further adjustment for gender, BMI, current smoker, physical activity, education, drinking, hypertension, diabetes, uric acid, ALT, TBIL, ALB, PLT, TG, TC, HDL-C, hs-CRP, and creatinine, the risk for NAFLD increased across the homocysteine quartiles, and the OR in the highest quartile compared with the lowest quartile was 2.08 (95% CI 1.61, 2.67). Similar results were also observed when homocysteine was considered as a continuous exposure variable (per SD increment, Table 2).

**Table 2:** Odds ratios for the association between serum homocysteine and the prevalence of non-alcoholic fatty liver disease

Homocysteine (μmol/L)	*N* = 7023	OR (95% CI)		
		Model 1	Model 2	Model 3
Quartiles				
Q1 (<5.1)	1876	1 (Ref)	1 (Ref)	1 (Ref)
Q2 (5.1 to <7.1)	1815	1.52 (1.30, 1.77)	1.44 (1.18, 1.74)	1.32 (1.04, 1.67)
Q3 (7.1 to <9.9)	1697	2.38 (2.04, 2.76)	1.90 (1.57, 2.30)	1.75 (1.39, 2.22)
Q4 (≥9.9)	1815	3.10 (2.67, 3.59)	1.91 (1.57, 2.32)	2.08 (1.61, 2.67)
*P* for trend		<0.001	<0.001	<0.001
Per SD increment	7023	1.47 (1.40, 1.55)	1.18 (1.10, 1.27)	1.25 (1.15, 1.37)
*P* value		<0.001	<0.001	<0.001

Model 1: adjusted for age^a^; Model 2: further adjusted for gender, body mass index, current smoker, physical activity, education, drinking; Model 3: further adjusted for hypertension, diabetes, uric acid, alanine transaminase^a^, total bilirubin^a^, albumin, platelet count, triglyceride^a^, total cholesterol, high-density lipoprotein cholesterol, high-sensitivity C-reactive protein^a^, creatinine.

Per SD increment represents 1 SD increment of log homocysteine.

^a^Variables were log transformed before analysis

### Subgroup analyses

**Fig. 1:**
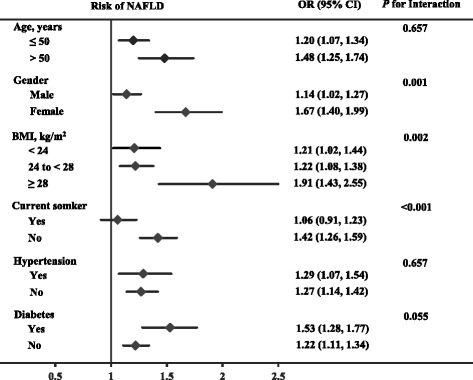
Association between homocysteine and non-alcoholic fatty liver disease in selected subgroups. Homocysteine is presented with a continuous scale (per SD increment of log homocysteine). Multiple logistic regression and interaction adjusted for age^a^, gender, body mass index, current smoker, physical activity, education, drinking, hypertension, diabetes, uric acid, alanine transaminase^a^, total bilirubin^a^, albumin, platelet count, triglyceride^a^, total cholesterol, high-density lipoprotein cholesterol, high-sensitivity C-reactive protein^a^, and creatinine. ^a^Variables were log transformed before analysis

To determine the effect of potential confounding factors, the associations between quartiles of homocysteine and NAFLD were further investigated among subgroups. As a result, we determined that the association differed significantly according to gender, BMI category and smoking status (*P* for interaction: 0.001, 0.002 and <0.001, respectively; Fig. 1). No effect modifications by age, hypertension, or diabetes on the association between homocysteine and NAFLD were observed (all *P* for interaction >0.05).

The subgroup analysis by gender indicated that homocysteine was strongly associated with NAFLD in females but was weaker in males (Fig. 1). After multivariate adjustment, the OR of having NAFLD with per SD increment of log homocysteine was 1.67 (95% CI 1.40, 1.99) in females, whereas the corresponding OR was 1.14 (95% CI 1.02, 1.27) in males. Similarly, obesity and non-smoking status were also related to an enhanced association between homocysteine and the prevalence of NAFLD (Fig. 1).

## Discussion

In this cross-sectional study, the main finding was that elevated homocysteine levels were positively associated with the prevalence of NAFLD in Chinese adults. Besides, in the subgroup analyses, an effect modification by gender, BMI and smoking on the association was found. A stronger association of homocysteine with the prevalence of NAFLD was observed in female, obese and non-smoking adults than in male, normal weight and smoking subjects.

Over the past decade, accelerating interest has developed in the relationship between homocysteine and the prevalence of NAFLD. On one hand, homocysteine is a sulfhydryl-containing amino acid mainly produced and catabolized in the liver [8, 9]. It is plausible that, in the presence of liver damage, alterations of serum homocysteine levels may occur. On the other hand, elevated homocysteine may conversely promote the progression of liver damage. Indeed, Yao et al. [23] recently found that hyperhomocysteinemia could promote hepatic steatosis in mice through activation of the aryl hydrocarbon receptor/CD36 pathway. According to several experimental studies, hepatic lipid accumulation was induced in different models of hyperhomocysteinemia [24–26]. In a study of pediatric NAFLD, Pastore et al. [27] found that homocysteine strongly correlated with the severity of liver damage. These previous studies suggested that homocysteine was associated with NAFLD progression. Meanwhile, accumulating evidence has demonstrated that homocysteine is an independent risk factor for cardiovascular diseases such as stroke [28, 29] and ischemic heart disease [30, 31], which suggests that homocysteine may mediate the association between NAFLD and cardiovascular diseases [32]. Thus, it is plausible that homocysteine might be an effective target for preventing NAFLD progression and its related cardiovascular complications.

Although several clinical studies have reported the association between homocysteine and the prevalence of NAFLD, the results were inconclusive. According to a case-control study including 50 patients with non-alcoholic steatohepatitis (NASH) and 30 healthy subjects, serum homocysteine levels were identified significantly higher in patients with NASH vs. controls [15]. Won et al. [33] found that the uppermost quartile of homocysteine in men was significantly associated with a 6.78-fold increased OR for NAFLD. Consistent with these findings, the positive association of serum homocysteine levels with NAFLD was shown in a meta-analysis by Dai et al. [34], which was based on eight studies (six cross-sectional and two case-control), totaling 935 participants (397 NAFLD cases and 538 controls). In contrast, another study suggested that serum homocysteine levels were not associated with NAFLD [35]. Furthermore, most related studies did not assess the independent risk of homocysteine with adjusting for potential confounders such as BMI, liver enzymes and dyslipidemia, and the relatively small number of subjects in these studies also made it difficult to draw definitive conclusions. The results of our study using a large sample with adjustment for multiple confounders support a positive association between homocysteine and the prevalence of NAFLD.

Our study also explored a possible effect modification by other related factors, such as gender and BMI, on the association between homocysteine and the prevalence of NAFLD. We concluded that the association was stronger in female, obese or non-smoking adults. In contrast to our study, a recent study found that homocysteine was closely associated with the prevalence of NAFLD in men but not in women [33]. However, only approximately 30 subjects with a small number of events were categorized in each quartile of homocysteine in that study, and it was hard to conduct a stable estimate for NAFLD. Interestingly, several previous studies have also suggested that serum homocysteine-related risk of cardiovascular diseases prone to be significant in women, but not in men [36–38]. Unfortunately, to date, the underlying mechanism of such gender-specific differences remains unknown. Obesity was reported to be associated with two key liver enzymes involved in homocysteine metabolism and may affect the catabolism of homocysteine in liver damage [39]. Our study firstly showed that the association between homocysteine and NAFLD was strengthened by obesity. Moreover, our study found that the association between homocysteine and NAFLD was attenuated in current smokers. Cigarette smoking is known to be associated with oxidative stress, the proinflammatory response, and fatty acid synthesis, which may have caused the phenomenon observed in our study [40, 41]. However, further studies are required to validate and explicate the effect modification noted in our study.

The present study has several limitations. First, this cross-sectional study was unable to explain the causal relationship; thus, further prospective cohort studies and intervention trials should be undertaken to establish a causal association between homocysteine and the prevalence of NAFLD. Second, the presence of hepatic steatosis was assessed by ultrasonography rather than liver biopsy pathology. Nonetheless, ultrasonography has been widely accepted for population-based studies due to its safety, economical cost and reasonable accuracy [21, 42].

## Conclusions

In conclusion, we found that homocysteine was significantly associated with the prevalence of NAFLD, particularly in female, obese or non-smoking adults. Further research is needed to determine whether the associations found in this study are causal and whether homocysteine-lowering therapy can be used to prevent progressive NAFLD and its related cardiovascular complications, particularly for female, obese and non-smoking adults.

## Abbreviations

ALT: Alanine transaminase
BMI: Body mass index
BP: Blood pressure
CI: Confidence interval
HDL-C: High-density lipoprotein cholesterol
hs-CRP: High-sensitivity C-reactive protein
NAFLD: Non-alcoholic fatty liver disease
OR: Odds ratio
TBIL: Total bilirubin
TC: Total cholesterol
TG: Triglyceride
